# Targeted Short Message Service-Based Intervention to Improve Routine Immunization Reporting in Bauchi State, Nigeria, 2016

**DOI:** 10.11604/pamj.supp.2021.40.1.15811

**Published:** 2021-12-23

**Authors:** Oluwasegun Joel Adegoke, Ester Mungure, Lynda Uju Osadebe, Olorunsogo Bidemi Adeoye, Matthew Aduloju, Idowu Makinde, Bakoji Ahmed, Patrick Mboya Nguku, Nadadilnasiya Endie Waziri, Peter Brian Bloland, Adam MacNeil

**Affiliations:** 1 African Field Epidemiology Network, Nigeria Country Office, Abuja, Nigeria,; 2 Global Immunization Division, Center for Global Health, United States Centers for Disease Control and Prevention, Atlanta, GA 30329, USA,; 3 Bauchi State Primary Health Care Management Board, Bauchi, Nigeria

**Keywords:** Routine Immunization, District health information system (DHIS2), Short message service (SMS), Reporting timeliness, completeness

## Abstract

**Introduction:**

High quality, timely and complete immunization data are essential for program planning and decision-making. In Nigeria, the National Health Management Information System (NHMIS) Routine Immunization (RI) module and dashboard (on the District Health Information System version 2 (DHIS2) platform) support the use of real time RI data. We deployed an automated short message service (SMS) notification system that works with the existing RI module to facilitate improvements in RI data in the DHIS2.

**Methods:**

A pilot project was performed using intervention and control local government areas (LGAs). A mixed methods approach using both qualitative and quantitative methods was used to evaluate the system. We assessed changes in reporting rates across different reports. The evaluation also included baseline and post-intervention surveys of health facility (HF) staff.

**Results:**

Reporting timeliness (76% pre and 99% post intervention) and completeness (83% pre and 99% post intervention) were consistently higher during the post-intervention than the pre-intervention period for facilities in the intervention LGA while reporting timeliness (65% pre and 66% post intervention) and completeness (71% and 77% post intervention) for facilities in the control LGA showed no change. Users reported that the SMS system was easy to understand and helped to facilitate improvements in consistency of data and timeliness of reporting. Inability of health care workers to effect changes at the HF level and the lack of immediate feedback were reported as key challenges to timeliness and quality of reports.

**Conclusion:**

An SMS-based intervention improved timeliness and completeness of health data reporting. However, the intervention should be evaluated on a larger scale over a longer time period before considering a national implementation.

## Introduction

In Nigeria, the National Health Management Information System (NHMIS) operates on the District Health Information System, version 2 (DHIS2) national instance platform [1]. This platform facilitates real-time access to aggregate health data. In 2014, a routine immunization (RI) module was introduced in Nigeria, starting with a pilot in Kano state. The RI module includes a dedicated RI dashboard that allows standardized visual monitoring of key immunization system indicators that are part of Nigeria´s routine immunization strategic and accountability framework [1,2]. The RI dashboard is available to government agencies and key stakeholders at all levels, creating an opportunity for data-driven decision-making within the immunization program.

Within Nigeria´s RI data reporting structure, health care workers (HCWs) are required to submit health facility-level aggregate data each month to the NHMIS and delays in reporting are common. Previous studies have shown that SMS reminders can improve attendance for hospital appointments and adherence to treatment [3,4]. A similar SMS approach targeting HCWs responsible for reporting RI data, if successful, could potentially speed up the identification and correction of data reporting problems, thereby better ensuring that reliable data are available for decision-making at the service delivery levels.

We developed an automated tool designed to work with the DHIS2 to send out targeted SMS on specific days in the month reminding HCW and the Local Government Area (LGA) officers to submit relevant reports. We implemented this tool as a pilot project to test the feasibility of using real time SMS notifications to improve timeliness and completeness of reporting RI data. Lessons learned from this pilot would also help provide evidence needed to assess the potential of this approach for wider, potentially national, implementation.

## Methods

### Selection of Intervention and Control LGAs

The pilot was conducted in Bauchi State in northeast Nigeria following the implementation of the DHIS2 routine immunization dashboard in the state. Bauchi State has 20 LGAs, 3 geopolitical zones (North, Central and South) and 1089 heath facilities (HF) of which 958 (88%) provide RI services [5,6]. Bogoro LGA (which has 29 HFs) was selected as the intervention site and Ningi LGA (with 71 HFs) was selected as the control site for this pilot. A desk review of data from November 2015 to April 2016 showed that these two LGAs ranked the lowest in two key reporting indicators (Table 1); completeness (the proportion of expected HF reports received) and timeliness (proportion of reports submitted on time). Four paper reports with vaccination data from each facility are entered intoDHIS2 at the LGA level each month by the LGA officer. The core NHMIS reports on the number of children that are vaccinated with each vaccine in the national schedule. The other three reports (vaccine utilization, NHMIS supplementary and microplan) were introduced during the implementation of the RI module. These reports contain additional vaccination data necessary for monitoring the immunization program, including the number of doses of inactivated polio vaccine (IPV) given, number of vials opened, number of vaccination sessions held, and the number of outreach and fixed sessions planned. Quantitative and qualitative methods were used to evaluate the effects of the automated SMS-based reminder system on reporting rate and determine the acceptability of the system among HCWs.

**Table 1:** description of the Intervention LGA and control LGA

Characteristics	Bogoro LGA (Intervention)	Ningi LGA (Control)
Percent Complete Report	83%	71%
Percent Timely Report	76%	65%
Total Number of HF	29	71
Percent of HF Offering RI	100%	100%
Percent of Public HF	100%	100%
Number of HF Administrator	29	71
Number of RI Focal Person	29	71
Number of M&E Officer	1	1
Number of LIO	1	1
Number of CCO	1	1

### Design and deployment of SMS system

**Figure 1:**
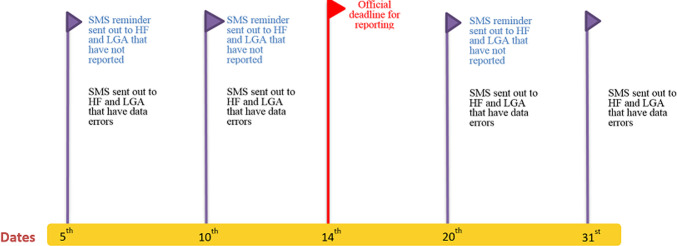
SMS system notification process

**Figure 2:**
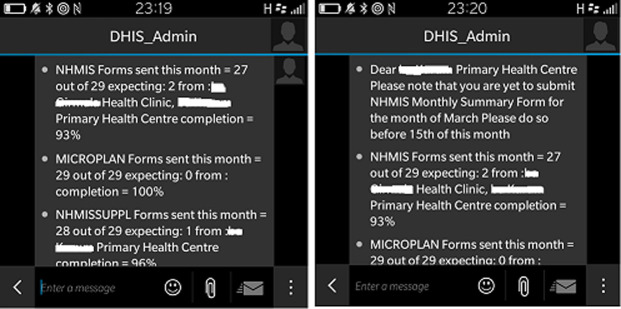
screenshot of sample SMS reminders

The SMS system was designed to extract and analyze data from DHIS2. The software is programmed in Java and is also Java Development Kit (JDK) Compliant and deployed on Tomcat™8 Server container. The database management system is MySQL running on a Linux Server. Using the DHIS2 National Instance Web Application Programming Interface (API) protocols, the customized SMS system pulled reporting rate data as a third-party software client. The Web API adheres to principles behind REST architecture. Based on established data validation rules, the customized system-generated SMS is sent to specific recipients on a given time and date (Figure 1), reminding them to submit the required RI reports (Figure 2).

Mobile telephone numbers for specified personnel at each HF in the intervention LGA were obtained and registered with the SMS application. At the HF level, the HF administrator (referred to as the HF in-charge) and the RI focal person were the primary recipients of the SMS messages. In general, the RI focal person has the responsibility of collating vaccination-related data, while the HF in-charge is responsible for verifying and approving all data sent from the HFs to the LGA. The SMS reminders were intended to prompt these staff to submit their HF monthly data to the LGA by the deadline.

At the LGA level, the LGA Immunization Officer (LIO), Monitoring & Evaluation Officer (M&E), and Cold Chain Officer (CCO) work as a team to monitor the timeliness and completeness of reporting from HFs and work to improve the accuracy of the LGA health data. These individuals also received SMS reminders from the system, summarizing completeness and timeliness (Figure 2) of the reports and providing a line-list of HFs yet to submit their data. The SMS were intended to remind these LGA officers to follow up with HFs yet to submit their RI reports for entry into the DHIS2.

### Health facility assessment

Timeliness and completeness of RI reports were two indicators selected for evaluating the intervention. Reports from all facilities offering RI for November 2015 to April 2016 were analyzed for both control and intervention LGAs in order to establish a baseline for completeness and timeliness. This process was repeated during the intervention phase from August 2016 to January 2017. The start-up phase of the pilot was between May 2016 to July 2016 and data for this period were excluded during the analysis. In addition, a field survey of 10 randomly selected HFs in each of the two selected LGAs was conducted between November 2015 and April 2016, covering human resources, data quality, data recording, infrastructure and capacity of health care workers to use RI data.

### Staff acceptability assessment

Qualitative data were collected through in-depth interviews of key informants, including state program officers (n=2) and LGA officers (n=3), and through a focus group of eight personnel from participating HFs. The data were used to determine acceptability of the SMS system among RI personnel and identify end-user challenges in the use of the SMS system for decision-making. To assess change in terms of human resources, self-reported data recording practices, infrastructure, and capacity at the HFs, the same questionnaire was used for the baseline assessment and the intervention assessment. The intervention questionnaire included additional questions regarding HCW´s perceptions of the acceptability of the system.

### Data management and analysis

We used Microsoft Excel for data management and the Statistical Package for the Social Sciences (SPSS) 16.0 for analysis. Reverse Kaplan Meier curves (Statistical Analysis System (SAS) 9.3) was used to create graphical representation of the time when the monthly reports were entered into DHIS2. Interviews and focus group data were transcribed using a template and analyzed using thematic method [7].

### Ethical considerations

Ethical approvals were obtained from Bauchi States´ Institutional Review Board and U.S. Centers for Disease Control and Prevention (CDC) for a non-research activity. Oral informed consents were obtained from participants in the focus group discussions and interviews. Written consents were obtained from HF respondents. Confidentiality was maintained throughout the assessment by using non-personal identifiers during data analysis.

## Results

**Figure 3:**
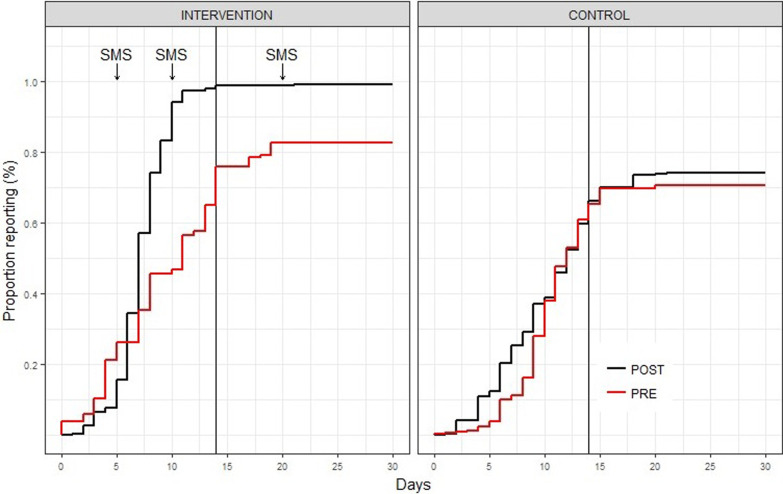
proportion of health facility (HF) reports for intervention (n=29 HFs) and control (n=71 HFs) LGA submitted in DHIS2. SMS were sent out on 5th, 10th and 20th of each month. The 14th of the month is the official deadline for timeliness and last day of each month for completeness

A total of 5,072 System-generated SMS messages were sent to HCWs during the intervention period (May 2016 - January 2017). Of the SMS sent, 4,909 (96.8%) were successfully delivered to the intended recipients. Based on a desk review assessment, there were no observed change before or after implementation in the intervention and control LGAs and their associated HFs in terms of human resources, self-reported data recording practices, infrastructure and capacity. The average timeliness of reporting in the pre-intervention period was higher in the intervention LGA (76 %) than in the control LGAs (65 %). The completeness of reporting in the pre-intervention period was also higher in the intervention LGAs (83%) than in the control LGA (71 %). In the intervention LGA, the average time to reporting was earlier compared to the pre intervention period 76% at pre and 99% at post intervention and a larger proportion of HFs reported compared to the pre-intervention period 83% at pre and 99% at post intervention. There were no observed changes in timeliness or completeness of reporting in the control LGAs (Figure 3).

Based on qualitative data, the SMS was reported to be easy to understand, and clearly specifying the HFs needing action and type of intervention needed, by all users at the state (n=2) and LGA (n=3) levels. The SMS contained information on completeness and timeliness of each paper report for each specific HF. Information provided through the notification was felt to be useful to the participants (n=8) in the intervention LGA in facilitating action. For example, the SMS sent to the LGA highlighted the names of HFs with pending reports, allowing for those HF to be immediately contacted for follow up.

Participants mentioned three main challenges to utilizing the SMS alerts for action: 1) the lack of authority of HF staff to make changes at their level; 2) lack of internet connectivity at the LGA level for data entry and accessing the RI dashboard; and 3) delays in LGA staff providing feedback to HF due to time constraints and competing priorities. Participants at all levels indicated the usefulness of the SMS system for improving reporting. Participants requested for the SMS system to be expanded to include additional indicators on data quality, such as comparison of co-administered vaccines and assessments of vaccine wastage. In addition, participants suggested getting feedback or confirmation on follow-up actions taken.

## Discussion

Data reporting from HFs in Nigeria is predominantly paper based. Studies in other sub-Saharan African settings show that the use of paper is associated with incomplete and inaccurate reports [8,9]. Completeness and timeliness were consistently high in the intervention group following introduction of the SMS system. These results suggest that this system was able to improve the timeliness and completeness of reported data. These results agree with similar studies that showed that interventions such as SMS have a potential to improve data reporting at the HF level, especially in low resource settings [3,8-10].

In the final survey, all HCWs interviewed expressed enthusiasm for the SMS system and felt that it encourages reporting in a timely manner. Competing demands for the time of LGA and HF staff were identified as a barrier to data use and follow-up action. In low resources settings with limited access to supportive supervision and funding, the use of phone calls, SMS messages and monthly review meetings offer a potentially affordable opportunity for providing feedback and coaching on data recording, reporting, and use. In Bauchi, LGA-level staff used the SMS alerts to guide development of action plans during review meetings and to prioritize which HFs to visit with their limited available resources.

Feedback and confirmation requested by HCWs could serve as an acknowledgment for job well done, indicating the value of incorporating such feedback system in future scale of the SMS project. Because of the SMS reminders, the LGA team changed data reporting procedures by bringing the reporting deadline for HFs forward to an earlier time and holding monthly review meetings earlier in the month. One major positive observation from this project is that the SMS system requires minimal ongoing technical assistance for maintenance after setup; apart from addressing programming bugs when identified and updating the database, the system is an automated process. Although not a part of this pilot, the system has the capability to provide monthly feedback to health facilities and instant update/alerts to the LGA and state teams.

Several challenges were observed during the pilot. We observed that some blank reports were submitted to the NHMIS, potentially due to increased scrutiny of the timeliness of reporting. During the initial weeks of implementation, a few programming problems in the SMS system were observed which resulted in some reminders not going out to recipients on schedule, however, these problems were rectified as they occurred. We also noticed the NHMIS national instance platform was occasionally offline, preventing the SMS system from extracting needed data. HF staff turnover posed a challenge since the system database needed to be updated periodically to ensure that there was always a responsible recipient to receive the reminders and notifications. This evaluation was designed as a pilot, so was not powered to demonstrate statistically significant impact. We had a small sample size and therefore were unable to determine if the effect we observed was due to chance. Finally, by increasing scrutiny of late reporting, it is possible that this approach may encourage HFs to falsify data in order to report on time.

## Conclusion

Although the size of the overall pilot and the number of interviews and surveyed HFs in the evaluation were relatively small, we found that use of a real-time SMS notification system improved completeness and timelines of reporting. However, because of the small size of this pilot, the intervention should be evaluated on a larger scale over a longer time before considering implementation on a national scale.

### What is known about this topic

- Short Message Service (SMS) is an approach used in generating reminders and providing feedback.

### What this study adds

- Use of targeted real time SMS notifications can improve timeliness and completeness of reporting Routine Immunization data.
